# Exploring the Influence of Sociocultural Factors on the Non-Utilization of Family Planning amongst Women in Ethiopia’s Pastoralist Regions

**DOI:** 10.3390/ijerph21070859

**Published:** 2024-06-30

**Authors:** Muluken Dessalegn Muluneh, Woldu Kidane, Virginia Stulz, Mhiret Ayele, Sintayehu Abebe, Andrea Rossetti, Gedefa Amenu, Azmeraw Ayehu Tesfahun, Makida Berhan

**Affiliations:** 1 Amref Health Africa in Ethiopia, Bole Sub City, Woreda 03, P.O. Box 20855, Addis Ababa 1000, Ethiopia; mihret.ayele@amref.org (M.A.);; 2 Faculty of Health, University of Canberra, 11 Kirinari St, Bruce, ACT 2617, Australia; virginia.stulz@canberra.edu.au; 3 Melbourne School of Population & Global Health, Melbourne University, Carlton, VIC 3053, Australia; 4 Amref Health Africa in Italy Via (St.) Volta n.10, Pregnana Milanese (Milan), 00198 Roma, Italy; andrea.rossetti@amref.it; 5 College of Agriculture and Natural Science, Debre Berhan University, Debre Berhan P.O. Box 445, Ethiopia; azmeraw2008@gmail.com

**Keywords:** family planning, nonuse, pastoralist, regression, sociocultural

## Abstract

This study aimed to explore the sociocultural determinants of family planning (FP) utilization among women in pastoralist areas of Ethiopia. A community-based cross-sectional survey was conducted involving 682 reproductive-aged women selected from three regions in pastoralist districts. Hierarchical logistic regression was used to identify factors associated with women who did not use FP. This study revealed that 47% of women did not use FP. Women who did not use FP were more likely to have shorter spacing between births, lack their partner’s support, not be involved in decisions regarding large household purchases, and have low household expenditures. Overall, the prevalence of not using FP is significantly high in pastoralist communities in Ethiopia. The authors recommend that investment in women’s health and FP be targeted at educational campaigns to raise awareness about FP and its importance. Engaging men and community leaders, promoting their support for FP and contraceptive use, and providing financial assistance to address financial barriers, such as transportation costs and healthcare fees, are important aspects that can increase the utilization of FP methods.

## 1. Introduction

Family planning (FP) has been identified as one of the essential reproductive health interventions and an essential array of commodities for achieving safe motherhood by reducing maternal and child mortality by limiting the desired number of children and increasing the spacing between births [1,2]. Globally, of nearly 2 billion women of reproductive age, 1.1 billion need to use FP, as there is a significant unmet need for FP among women, and of these women, 842 million are users of modern methods of contraception [3]. Although modern FP usage is rampantly increasing across the world, there is still a high unmet need for modern contraceptives in many developing countries. The data reveal that a large proportion of women who require FP services are not accessing them. This gap highlights a critical issue that needs to be addressed to ensure that women’s reproductive health needs are met.

In sub-Saharan Africa (SSA), the utilization of contraceptive methods is one of the lowest in the world [4]. It has also been predicted that preventing barriers to the use of contraceptive methods and reaching all women with unmet needs can prevent the 74 million unintended pregnancies, 25 million unsafe abortions, and 47,000 maternal deaths occurring each year in low- and middle-income countries [5].

Similar to global trends, in Ethiopia, the use of contraceptives has been increasing over the past two decades; however, evidence has indicated that 4.5 million women of reproductive age have an unmet need for modern contraception [6]. According to the 2016 Ethiopian Demographic and Health Survey (EDHS), there is a high unmet need for contraceptive methods in Ethiopia compared to the target of 10%, which might be due to ongoing conflict, drought, and the effect of COVID-19 [7]. In addition, there is a large difference in the FP indicators between the pastoralist and agrarian regions of Ethiopia. The unmet need for FP is significantly high in pastoralist areas of Ethiopia [8]. According to the 2016 Mini EDHS report, the prevalence of modern contraceptive methods in the Afar Region, Oromia, and the Southern Nations, Nationalities, and Peoples’ Region (SNNPR) was 12%, 28%, and 40%, respectively, which is far below that in Addis Ababa (50%) [9,10]. Despite the tremendous efforts over the years towards improving FP utilization and reducing the unmet need for FP, still, more than one in five currently married Ethiopian women (22%) still have an unmet need for FP. Oromia, Afar, and the SNNPR are among the top regions with a high unmet need for FP [9].

Pastoralist communities are social groups whose way of life is centred around herding livestock, typically in nomadic or seminomadic settings, and relies on domesticated herbivores for sustenance and resources. These communities play significant roles in contributing to their respective regions by driving economic activity, enriching cultural diversity, and promoting environmental sustainability [11,12]. These communities depend on raising livestock for their living. By trading livestock and related goods, they boost the local economy [10]. They cherish unique traditions and practices, which contribute to the diverse culture of the regions. Their ceremonies, rituals, and knowledge about raising livestock are deeply rooted in their heritage [12].

The pastoralist lifestyle in Ethiopia is deeply rooted in cultural practices and beliefs that have passed through generations, and these beliefs often conflict with modern FP methods. It is therefore timely to dive deep into the current cultural and social norms that affect the use of modern FP methods [13]. Evidence in other countries also highlights the influence of sociocultural and religious contexts on the utilization of modern contraceptive methods. For instance, in Kenya, fear of side effects, such as having a baby with a deformity, weight gain, and infertility, have been found to be positively associated with not using modern contraceptives [14]. Similarly, studies in Mali also indicated that many women feared that the pill and injectables could cause permanent infertility [15].

Various studies conducted in pastoralist areas in other countries have shown that religion, literacy, income status, occupation or employment status, and a desire to have more children are significantly associated with FP utilization and sociocultural practices [13,16,17,18,19,20]. In the pastoralist context of Ethiopia, there are deeply rooted cultural and social norms that often clash with modern FP practices, and limited research has been conducted on FP utilization, with only a few small-scale studies available. Moreover, some existing studies are outdated, lacking current data and insights into contemporary challenges, and have been conducted in small pocket areas within pastoralist populations. This highlights the urgent need to explore these sociocultural factors in greater depth. Therefore, this study aims to fill this gap by providing detailed insights into the sociocultural factors affecting FP utilization in pastoralist communities by providing up-to-date information that reflects the current sociocultural and economic landscapes of pastoralist regions in Ethiopia by incorporating multiple pastoralist contexts in the country. In addition to examining factors directly related to FP use, this study adopts an intersectional lens to explore how intersecting structural factors (e.g., access to healthcare, socioeconomic status) shape FP decisions. This intersectional analysis allows for a more comprehensive understanding of the complex dynamics underlying FP practices in pastoralist contexts. The objective of our study was to investigate the key sociocultural determinants of FP utilization among women in pastoralist areas of Ethiopia.

## 2. Materials and Methods

### 2.1. Study Design and Study Setting

A community-based cross-sectional study was conducted in three regions and six districts to uncover the underlying cultural beliefs and practices related to modern FP utilization and to characterize the current status in pastoralist communities in Ethiopia in January 2024. Ethiopian pastoralist communities are primarily found in the lowland areas of the country, particularly in the regions of Oromia, southern Ethiopia, and Afar. These regions are known for their diverse pastoralist groups and their traditional way of life, centred around livestock herding. In southern Ethiopia (South Omo), pastoralist groups such as Hammer and Dasenech reside in scattered pastoralist settlements along the river valleys and grasslands. Similarly, in Oromia (Borena), pastoralists are found primarily in the Arero districts, while in Afar (Zone 1), communities inhabit areas such as the Afambo and Assayata districts. The pastoralist communities in Borena, Afar, and South Omo in Ethiopia are known for their traditional pastoralist way of life, where they rely on livestock for their livelihoods. The Borena and Afar pastoralist communities are known for their resilient and adaptive survival strategies. However, these pastoralist communities face various challenges, such as land degradation, water scarcity, and conflicts over resources with other communities that threaten their way of life and livelihoods. There have been efforts to support and promote the sustainable development of these communities through various initiatives, although they were not as successful as intended. This study was conducted in pastoralist communities in three regions: southern Ethiopia (Hammer and Dasenech); Oromia (Borena)—Arero district; and Afar (Afambo and Assayta districts).

### 2.2. Sample Size and Sampling

A single population proportion formula was used to estimate the sample size, assuming a 95% CI and 5% margin of error, by taking a 50% proportion of the FP utilization status assuming an unknown population in the pastoralist community, a design effect of 1.55, and a non-response rate of 15%. Accordingly, the final calculated sample size was 682 reproductive-aged women. The sample size was proportionally distributed in six randomly selected districts in three regions. These included (i) southern Ethiopia (South Omo zone): Hammer, Dasench, and Gnagatom; (ii) Oromia (Borena)—Moyale, Dhaas, and Arero districts; and (iii) Afar (Zone 1)—Afambo, Assayta and Elidar districts. Multistage sampling techniques were employed to select districts, and systematic sampling procedures were employed to reach study participants for whom actual data were collected. The women were identified from the sampling frame, which was generated through the assistance of health extension workers stationed at nearby health posts. A systematic approach was employed to select women from the registered sampling frame.

### 2.3. Data Collection Methods

A pretested structured survey was adapted from the Ethiopian Demographic and Health Survey (EDHS) and previous surveys that were designed to explore factors related to FP utilization barriers. A structured survey was administered to women in the community. Quantitative data for the study were collected electronically using the mobile applications ODK/KOBO, where a structured survey with precoded answers was uploaded. The tools for the data collection were developed in consultation with people who know the culture and language of the study community, and surveys were pretested before actual data collection. The quantitative variables were assessed against established standard measurement scales, helping to anchor the data in objective, validated metrics. We carefully checked the distribution of these quantitative variables to confirm the homogeneity of the data distribution across the sample. Once the variable distributions were validated, we categorized the quantitative variables based on the standard measurement thresholds. This categorization process facilitated an easier comparison of the sample data against the established standards. Additionally, to enable more robust statistical analysis like logistic regression, we recoded the categorical responses into dummy variables.

In this study, a total of 682 copies of the questionnaire were administered across 3 regions, as presented in Table 1, as follows: (i) in the Afar region, 251 questionnaires; (ii) in the Oromia region, 108 questionnaires; and in the southern Ethiopia region, 323 questionnaires were administered. The questionnaire contained a total of 48 items/questions for each participant.

We carefully considered sampling in the analysis to avoid potential bias. For instance, to reduce the potential bias in this study, we used a pretested questionnaire, which is consistent for all three regions, and the sample was randomly selected within each region to obtain a representative sample, rather than relying on a biased convenience sample. The data were collected electronically to avoid potential individual bias, and data were validated through validation checks and statistical analyses to identify and account for any systematic differences between the regional samples.

### 2.4. Data Analysis

Both descriptive and inferential data analysis techniques were employed to analyse the data collected using quantitative tools in STATA version 18. Logistic regression methods were employed for this study. To enable logistic regression, the responses were recoded to dummy variables. While initial bivariate analyses provided insight into the relationship between individual sociodemographic variables and FP utilization, multivariate analysis enabled us to understand the combined influence of multiple factors. By considering interactions among variables, we identified the most influential factors and obtained a more accurate understanding of their impact on FP utilization.

## 3. Results

### 3.1. Demographic Characteristics of the Respondents

The survey included a total of 682 women in three regions of Ethiopia, 47% of whom were from southern Ethiopia based on the size of the population. The majority of respondents were illiterate (86%), with only 8% completing primary education. The religious composition was predominantly Muslim (42%), followed by nonbelievers (19%) and traditional belief followers (21%). The mean age of the respondents was 29.1 years. The majority of participants were married or living together (72%), with the majority reporting arranged marriages (89.25% women). Approximately one-fifth of the respondents married before turning 18 years of age. Almost half (47.26%) of the women reported that their husbands had other wives or partners.

### 3.2. Socioeconomic Characteristics of the Study Participants

The primary sources of income of many of the respondents (80.5%) were primarily subsistence agriculture (56.2% from animal husbandry and 24.3% from smallholder land cultivation). Less than 5% of the respondents reported paid jobs as their primary source of income. Additionally, less than one-third (32.7%) of the women respondents stated that they were currently working. The average monthly household expenditure of women respondents was reported to be 1940.3 Ethiopian Birr (ETB) (1 USD = 56.5 ETB), with the minimum being ETB 120 and the maximum being ETB 8500. However, the average monthly household income was reported to be ETB 2794.7, with the minimum being ETB 200 and the maximum being ETB 35,000.

### 3.3. Fertility, Unwanted Pregnancy, and Contraceptive Practices

The results of the survey regarding fertility and contraceptive practices revealed that the majority of respondents had given birth (70%), with almost two-thirds (61.2%) having a birth spacing of less than two years. Eighty-one percent of the respondents had desired their last pregnancy (index pregnancy), indicating a planned pregnancy, while 19.08% had not. Among the reasons for unwanted pregnancies, more than one-third of the respondents (39%) mentioned that they did not know how to delay pregnancy at that time, and 11% of the respondents did not know how to access the service at that time, while almost a quarter of the respondents (24%) reported religious prohibition of pregnancy prevention measures. One-fifth of the respondents also mentioned that their husbands had forced them to become pregnant. Almost two-thirds (64%) of women respondents wanted to have more children, while 17% of them did not want to have more children. Fewer than 5% of the respondents reported that they could not determine the number of children to have, and just over a tenth (11%) of them were not sure about it. More than half (57%) of the women respondents wanted to have their next child in less than two years, while 27% of them wanted to give birth between two to four years.

### 3.4. Contraception Use, Knowledge, and Barriers

The results of the study indicated that contraceptive uptake among women in the intervention areas was relatively low in comparison to that in other regions of the country. Almost half (47.07%) of the respondents had never used contraception in their life, and 52.93% of the respondents reported that they had used contraception before to delay pregnancy. Among those women respondents who used contraceptives, almost half (49.63%) were currently using a contraceptive method, while half (50.37%) of the total women respondents were not currently using any FP methods. The most commonly used contraceptive methods in the study areas were found to be injectables (54.14%), followed by implants (51.78%), pills (34.62%), IUDs (14%), condoms (2%), and other methods (2.9%). The percentage of respondents with contraceptive knowledge (at least one method) in this study was 98%, which is similar to the national figure (>99%). In terms of knowledge of contraception methods, injectables and implants were the most commonly known methods, followed by pills and male condoms. This study indicated that approximately 62.61% of respondents reported that they used to visit health facilities for family-planning-related services, while almost two-fifths of the respondents (37.39%) did not.

### 3.5. Reasons for Discontinuation of FP Services

**Figure 1:**
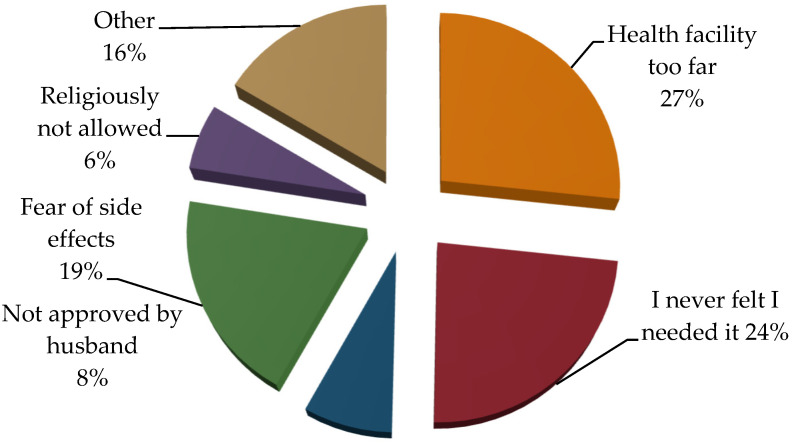
Reason for discontinuation of FP services (*n* = 236).

The findings of this study showed that the discontinuation of contraceptive use was influenced by various factors. Among the respondents with a fear of side effects (19%), 27% reported that the primary reason for discontinuing contraceptive use was travelling long distances to health facilities, which made it challenging to access necessary services and supplies. Additionally, 24% of the respondents mentioned that they had never felt the need to continue using contraceptives, possibly due to changes in their reproductive plans or a lack of awareness about the importance of consistent contraceptive use. This study also revealed that 8% of respondents cited disapproval from their husbands or mothers-in-law as a major reason for the discontinuation of contraceptives. Fear of side effects was mentioned by 19.44% of respondents, suggesting concerns about the potential negative impacts of using contraceptives. Furthermore, 6% mentioned that their religious beliefs did not allow continued contraceptive use (Figure 1).

### 3.6. Attitudes towards FP and Reproductive Rights of Women

The results of this study regarding the perspectives of individuals about pregnancy and its impact on families revealed that more than half (54%) of the women highlighted the positive impact of pregnancy prevention on the economic stability of families, and 22% of women recognized the health benefits of preventing pregnancy. Of the women surveyed, 44% completely agreed that they should suggest contraceptive use with their husbands, 25.8% of women slightly agreed that it is acceptable for wives to suggest contraceptive use to their husbands, and less than half (46%) indicated that their husbands do not support the use of modern contraceptive methods. When asked about their agreement with whether wives should suggest contraceptive use to their husbands, a significant percentage (43%) completely or slightly agreed (28%) that it is normal, while a lower percentage of the respondents (17%) disagreed on the issue. This suggests that pastoralist women should be supported by their husbands or partners in family-planning-related issues. Similarly, almost two-thirds (65.5%) of the respondents indicated that the community disrespected unmarried women if they used FP services. Similarly, more than two-thirds of the respondents (68%) mentioned that unmarried women ceased using FP methods because of fear of disrespect from the community.

More than two-thirds (69%) of husbands agreed that they expected their wives not to use FP services so that they could have large families. In particular, almost three-quarters (71.5%) of women respondents reported that they were expected not to use FP services so that they could have large families. Less than a fifth (19%) of the respondents disagreed with this expectation (see Table 2 below).

The results of the survey regarding women’s decision power indicated that they have limited decision power even to exercise their sexual reproductive health and rights. The survey revealed that more than two-thirds (70%) of the respondents believed that conflicts would arise if married women used FP methods without the consent of their husbands, potentially leading to a divorce. Only approximately 18% of the respondents disagreed with this viewpoint, and 11% responded that they were unsure about the consequences. In relation to this, more than two-thirds (68%) of respondents mentioned that married women usually stop using FP due to fear of conflict with their husbands.

Consistent with this, almost two-thirds (61%) of the respondents believed that a man should never allow his wife to decide on the size of the family. Similarly, more than two-thirds (68%) of the respondents reported that it was mainly women’s responsibility to ensure that contraception was used regularly.

Almost half (43%) of the respondents reported not having engaged in open discussions about FP with their partners.

The results of this study also highlighted that more than half (58%) of the respondents agreed that a girl should never get married before 18 years of age, although one-fifth of the respondents still believed that a girl should get married when the father/husband decided, irrespective of her age. Likewise, 58% of the respondents believed that a girl should never give birth before 18 years of age, while one-fifth of the respondents believed that a girl could give birth anytime she thought it best for her. Almost one-third (28.9%) of the respondents also believed that unmarried women under the age of 18 should not be allowed to use FP services, while 58.74% agreed that they should be allowed to use FP services. In terms of birth spacing, more than half of respondents (52.6%) preferred a gap of less than two years between births, 27% preferred a gap of two to three years, and a smaller percentage (11%) had other preferences. A small proportion of the respondents (9.24%) were undecided or did not know about their preference for birth spacing.

### 3.7. Women’s Decision-Making Skills

The results on gender roles and decision-making within a household revealed that more than two-thirds (70.33%) of respondents agreed that it was mainly the women’s responsibility to ensure that contraception is used regularly, although almost a quarter (22.03%) of the respondents disagreed (see Table 3). Almost two-thirds (60.5%) of the respondents agreed that a man should never allow his wife to decide on the number of children to have. Likewise, almost one-third (31.23%) of the respondents believed that the husband should take the upper hand in making decisions on large household purchases, while 54.55% believed that it should be a joint decision made by both the wife and husband.

In terms of healthcare decisions for women, almost three-quarters (71.7%) of respondents believed that it should be a joint decision made by both the wife and husband. A smaller percentage (17.3%) thought the husband should make this decision, while only 1.32% believed the wife should make this decision. Similarly, almost three-quarters (71.11%) of respondents believed it should be a joint decision made by both the wife and husband on the use of contraceptives. Again, a smaller percentage (17.3%) thought the husband should make this decision, while 3.81% believed the wife should make this decision.

### 3.8. Multivariable Binary Logistic Regression

Hierarchical logistic regression was fitted to sequentially add groups of variables to the model without using FP as the dependent variable. Predictor variables were grouped based on their conceptual similarities, such as sociodemographic situation, socioeconomic situation, access to and use of FP, knowledge, attitudes, and perceptions, and, finally, sexuality and gender norms. The final model was selected because of its better fit to the data. The likelihood ratio chi-square test statistic was 263.06, with a *p*-value of 0.0000, indicating that the model significantly predicted the outcome variable. Moreover, the pseudo-R-squared value was 0.4382, suggesting that the model explained approximately 44% of the variance in the outcome.

The analysis revealed that the odds of not using FP were approximately threefold greater for individuals with a birth spacing of less than two years than for those with longer birth spacing. This odds ratio is statistically significant, with a *p*-value of 0.002, suggesting that the relationship between birth spacing and not using FP was unlikely to be due to random chance. In addition, the odds of not using FP were approximately 7.7 times greater for individuals who did not plan their last pregnancy than for those who did want a pregnancy for the index child. It is also significant, with a *p*-value of 0.000, indicating that the relationship between not wanting the pregnancy for the index child and not using FP is unlikely to be due to random chance.

Moreover, compared to women who had their husbands’ support when taking contraception, women whose husbands did not support this were approximately 16 times more likely to not use FP (AOR = 15.88), with a *p*-value of 0.000. This odds ratio is statistically significant and suggests that there is more reason than chance for the association between not using FP and a husband’s lack of support. Thus, the logistic regression analysis showed that women who did not receive spousal support for contraceptive use were far more likely to not use FP than those who did. The likelihood of not using FP was approximately two times greater for women who did not think that it was mainly the woman’s responsibility to ensure contraception use than for those who thought it was the woman’s responsibility. This odds ratio (AOR = 2.00) is statistically significant, with a *p*-value of 0.047, suggesting that the relationship between this belief and not using FP is unlikely to be due to random chance (Table 4).

The results of the multiple logistic regression analysis also revealed a significant association between the husband’s decision-making authority for large household purchases and the likelihood of not using FP methods. Specifically, the odds ratio of 0.176, with a *p*-value of 0.000, indicated that when a woman does not have decision-making authority on large household purchases or if only a husband or someone else decides on these purchases, the odds of not using FP methods increased by approximately 82.43% in comparison to when she was involved, holding all other variables constant. Similarly, the analysis also indicated a statistically significant association between lower decision-making authority for her own healthcare services and the likelihood of not using FP methods. The odds ratio of 3.54, with a *p*-value of 0.005, suggested that when a woman has no decision-making authority or when the decision-maker is limited to a husband or someone else, the odds of not using FP methods increased by approximately three times compared to when the woman has decision-making authority for healthcare services. This emphasizes the need to consider the role of decision-making authority as a crucial factor in influencing FP behaviour. These findings provide valuable insights for further research and initiatives focused on understanding the complex interplay between household dynamics and FP practices (Table 4).

We hypothesized that sociocultural factors would be critical for the nonuse of FP, as these factors are deeply rooted in these communities. However, our multiple regression analysis found that while sociocultural factors were important, the barriers to the nonuse of FP were more complex, involving an interplay of individual, social, and other factors. This suggests that the factors influencing FP utilization in these pastoralist communities are multidimensional and go beyond just sociocultural determinants. To better understand this complexity, we recommend applying an ecological model to analyse the interplay of factors at different levels (individual, interpersonal, community, and societal). This holistic approach could provide valuable insights to inform decision-makers and program designers on how to effectively improve FP access and utilization among women in pastoralist areas.

The amount of household expenditure is one of the significant predictor variables, with an odds ratio of 0.90 and a *p*-value of 0.018. Despite the odds ratio being close to 1, indicating a minimal effect, the statistically significant *p*-value provides evidence that lower levels of expenditure do have a discernible impact on the likelihood of not using FP methods. Even though sociocultural factors are critical for the nonuse of FP, other barriers were more complex, such as individual, social, and other factors.

## 4. Discussion

Our study assessed the sociocultural impact on women’s utilization of modern contraceptives in pastoralist areas of Ethiopia. The results of our study revealed that 47% of the overall population did not use FP, with regional variations reported. The findings of our study are lower than those of the Mini EDHS 2019, which reported that overall, 88% of Afar, 72% of Oromia, and 60% of the SNNPR did not use FP [9]. This variation might be due to changes in sociocultural factors, improved accessibility of healthcare services, increased availability and awareness of contraceptive methods, and evolving patterns of reproductive health behaviours over time that might increase the use of FP.

The findings of our study showed that there was a statistically significant association between decreased birth spacing, lower expenditure, and low decision-making authority in women who did not use FP methods. Women who reported that they had shorter (less than one year) birth spacing were three times more likely not to use FP than those who had a birth spacing of two or more years. This finding is supported by a systematic review and meta-analysis [21]. Our study showed that women who had never used contraception before the conception of their last child were 3.87 times more likely to have shorter birth spacing than women who utilized contraceptive methods. This can be explained by living in pastoralist communities and cultural or religious beliefs that prioritize fertility or discourage modern contraceptive use. Additionally, a strong preference for larger family sizes and societal norms encouraging frequent childbearing can contribute to shorter birth intervals, as women may not actively seek to delay or space pregnancies. This might also be related to lower awareness about FP, religion, and limited access to family services [21,22]. Moreover, the potential for the perception of affordability might also contribute to these areas; although the services provided in Ethiopia are free, the indirect cost of transport can contribute to limited access [23].

Our study revealed that lower household expenditures were associated with not using FP. Our study showed that an increase in monthly income was associated with the likelihood of using FP methods. Mothers who had higher monthly incomes had greater odds of using FP than the mothers with the lowest incomes. This might be due to limited household expenditure impacting access to healthcare facilities and services. Families with lower expenditures may face barriers such as transportation costs, healthcare fees, or a lack of nearby facilities offering FP services, further hindering the use of FP.

Moreover, women whose husbands or someone else (other than themselves) made decisions about large household materials (82%) were significantly less likely to use FP than women who make these decisions themselves or jointly with their husbands. On the other hand, when decisions about women’s healthcare were made solely by their husbands or someone else, women were 3.4 times less likely to use FP in comparison with women who made decisions by themselves or jointly. The findings of our study are supported by a community-based cross-sectional study conducted in pastoralist communities in Ethiopia [17]. Our study revealed that women’s increased decision-making about FP is significantly associated with increased FP use. In pastoralist community contexts, women’s decision-making autonomy reflects broader shifts in gender norms and power dynamics within households and communities. Increased involvement in decision-making can signify progress towards gender equality and women’s empowerment [23].

In our study, women who disagreed with the idea that it is mainly women’s responsibility to ensure that contraception is used regularly were found to be twice as likely not to use FP in comparison to women who agreed with this responsibility. This may be because women who believe that contraception is primarily their responsibility are also more likely to take ownership of FP decisions and actions. When women do not see contraception as their responsibility, they may be less proactive in seeking and using FP services [24].

Women whose husbands do not support the use of modern contraceptives are 16 times more likely to not use FP than women whose husbands support modern contraceptive use. To explain this, the absence of spousal support for modern contraception significantly influences women’s decision-making regarding FP practices. This finding is supported by a community-based cross-sectional study conducted in the Bale Zone [16]. Our study showed that the odds of modern contraceptive utilization among women whose husbands supported the use of modern contraceptives were eight times greater than those among women whose husbands did not support the use of modern contraceptives. In pastoralist societies, husbands often hold significant decision-making power within relationships. Cultural or religious beliefs that prioritize large families or oppose contraceptive use can influence husbands’ attitudes towards FP. If husbands oppose modern contraceptive use, women may face difficulties using FP methods. Women may conform to their husbands’ beliefs or preferences due to societal norms or religious teachings, impacting their decision-making on using contraceptives [25].

Women who experienced unwanted pregnancies were 7.2 times more likely not to use FP than women who wanted or planned a pregnancy. Our study is supported by a study in Jimma, which revealed that unintended pregnancy was strongly associated with not using contraceptives [26]. This suggests a gap in contraceptive access, knowledge, or utilization.

The findings reported in this study provide vital evidence to inform policy and guide health practitioners in responding to and increasing FP uptake, aligning with the SDGs. These results offer a helpful lens through which program designers and decision-makers can understand the multifaceted nature of the issue and craft appropriate interventions. A key implication is the need for a broad, multi-sectoral approach that is backed by vigorous legal enforcement systems. For instance, at the program level, providing financial assistance or incentives to economically disadvantaged households could improve their access to healthcare services, including FP. Addressing financial barriers such as transportation costs and healthcare fees may help increase FP utilization among these populations. At the policy level, it will be critical to ensure that the financial hardships faced by women are mitigated and to create educational opportunities and safeguard the basic rights of women with equity, particularly in pastoralist communities in Ethiopia. This integrated, equity-focused approach across various sectors is essential to effectively prevent and respond to violence in alignment with the SDG targets. Overall, the findings of this study present a crucial evidence base to guide policymakers and practitioners in developing impactful, multifaceted strategies to address this complex social challenge and work towards the 2030 SDG aims.

### Limitations of the Study

Although great care was taken in the respondents’ recruitment, and they were provided training about the content of the questionnaire, there were unavoidable individual differences that seemed to have influenced how the respondents replied to some of the questions. Furthermore, this study only focused on quantitative and cross-sectional studies. Therefore, qualitative findings were not included, which may provide further information on the barriers to FP use. A cross-sectional study does not show causation.

## 5. Conclusions and Recommendations

Overall, our study highlights the complex interaction between sociocultural, economic, and gender dynamics that influence FP practices in pastoralist communities in Ethiopia. The proportion of women not using FP was significantly high, which is an indication of the high unmet needs of women’s reproductive health. Most importantly, our study revealed that a lack of partner support significantly contributed to not using FP methods among women. Additionally, the study revealed that women who do not have high decision-making authority on large household purchases or healthcare services are also more likely not to use FP methods, underscoring the importance of women’s empowerment and autonomy in reproductive health decisions. Moreover, our study revealed that lower household expenditures are associated with not using FP, indicating that financial barriers, such as transportation costs and healthcare fees, can hinder access to FP services. Furthermore, our study also emphasizes the meaningful connection between limited birth spacing and not using FP services, suggesting that cultural or religious beliefs that prioritize fertility or discourage modern contraceptive use may contribute to shorter birth intervals. This study contributes to knowledge and understanding of the current state of nonuse of FP and provides insights into factors that contribute to nonuse, with a particular focus on pastoralist communities. Additionally, this study helps programmers and decision-makers to focus targeted investment on women’s health in general and FP in particular. The findings from this study underscore the need for a comprehensive, context-specific strategy that goes beyond targeting sociocultural factors alone. Investing resources to address the multifaceted barriers to FP in these communities is crucial for improving women’s health outcomes.

Based on the findings, the following recommendations are proposed for policy and program implementation: (I) Implement targeted educational campaigns to raise awareness about the importance of FP, birth spacing, and contraceptive methods. These programs should focus on dispelling myths, addressing cultural beliefs, and promoting the benefits of FP for women’s health and well-being. (II) Encourage women’s involvement in decision-making processes, which can enhance their autonomy and lead to the increased uptake of FP methods. (III) Establish programs that engage men and promote their support for FP and contraceptive use and the benefits of FP. Addressing misconceptions and involving them in FP decisions can positively impact women’s access to the utilization of contraceptive methods. (IV) Provide financial assistance or incentives to households with lower expenditures to improve access to healthcare services, including FP. Addressing financial barriers such as transportation costs and healthcare fees can increase the utilization of FP methods among economically disadvantaged populations. (V) Engaging community leaders, religious figures, and local influencers can help challenge societal norms, reduce stigma about FP, and encourage positive attitudes towards contraceptive use.

By implementing these policies based on the research findings, Ethiopia can work towards improving FP practices, empowering women, and enhancing reproductive health outcomes in pastoralist communities.

## Figures and Tables

**Table 1:** Characteristics of the pastoralist communities.

Variable	Category	Women	
		*n* = 682	%
Region	Afar (Assayita and Afambo)	251	36.8
	Oromia (Arero district)	108	15.84
	Southern Ethiopia (Hammer and Dasenech)	323	47.36
Education	Illiterate	586	85.8
	Primary between (1 to 8)	54	7.91
	Low secondary between (9–10)	22	3.22
	Preparatory between (11–12)	2	0.29
	Technical/Vocational	2	0.29
	Higher Education	16	2.34
Religion	Muslim	285	41.73
	Traditional, including Wakefeta	174	20.94
	Nonbeliever	122	17.86
	Christian Protestant	49	7.17
	Christian Orthodox	35	5.12
	Catholic	17	2.49
Marital status	Married	427	62.52
	Living with a partner	66	9.66
	Single	159	23.28
	Widowed	14	2.05
	Separated	11	1.61
	Divorced	5	0.73
Age (Mean)	Mean	29.1	
Age at first marriage	18 years and above	545	79.92
	Less than 18 years old	137	20.08
Marriage Arrangement	No, it is on my will	73	10.75
	Yes, it was arranged	609	89.25
Besides yourself, does your (husband/partner) have any other wives/partners?	No	260	52.7
	Yes	233	47.3
How many other wives or partners does your partner have?	Only one	163	69.96
	>2 wives	70	30.1

**Table 2:** Attitudes towards the FP and reproductive rights of women.

Variable	Category	Respondent	
		*n* = 682	%
Do you think that prevention of pregnancy is good for a family?	Yes, it is good for the family economy	365	53.5
	Yes, it is good for the health of the mother	148	21.7
	No, it is not good in relation to culture, religion and family	122	17.9
	Yes, it is good because for other reasons	47	6.89
Do you think that it is normal that a wife/partner can suggest to her husband that they use contraceptives?	Completely Agree	298	43.63
	Slightly Agree	176	25.77
	Not Sure	88	12.88
	Slightly Disagree	73	10.69
	Strongly Disagree	47	6.88
Do you think the community disrespects her if unmarried women use FP services?	Completely Agree	447	65.45
	No, Disagree	129	18.89
	Not Sure	105	15.37
Unmarried women cease using FP methods because of fearing disrespect from the community.	Completely Agree	462	68
	Not Sure	80	12
	Disagree	140	21
Husband supports the use of modern contraceptive	Yes	370	54
	No	312	46
Most husbands highly expect their wives not to use FP services so as to have large family size	Slightly Agree	258	37.77
	Completely Agree	231	33.82
	Not Sure	73	10.69
	Slightly Disagree	73	10.69
	Strongly Disagree	47	6.88
If married women use FP method without the consent of their husband’s conflict will even end in divorce	Completely Agree	289	42.31
	Slightly Agree	197	28.84
	Slightly Disagree	87	12.74
	Not Sure	72	10.54
	Strongly Disagree	37	5.42
Do you believe that a man should never allow his wife to decide on the size of the family?	Completely Agree	237	34.7
	Slightly Agree	175	25.62
	Slightly Disagree	150	21.96
	Strongly Disagree	62	9.08
	Not Sure	57	8.35
Do you make open discussion with your partner about FP?	Yes	382	56.39
	No	300	43.61
In your opinion, what is the ideal age a girl should get married?	Never before 18 years old	417	61
	When the father/husband decides	166	24
	Anytime she thinks is the best for her	52	8
	Before 18 years old	38	6
	Other (specify)	9	1
According to you, what is the ideal age a girl should give birth for the first time?	Never before 18 years old	395	58
	Anytime she thinks is the best for her	138	20
	When the father/husband decides	71	10
	Before 15 years old	39	6
	Before 18 years old	30	4
	Other (specify)	9	1

**Table 3:** The decision-making power of women in pastoralist communities, Jan 2024.

Variable	Category	Frequency (%)	
		*n* = 682	%
Do you think that it is mainly the woman’s responsibility to ensure contraception is used regularly?	Completely Agree	255	37.34
	Slightly Agree	224	32.8
	Slightly Disagree	89	13.03
	Strongly Disagree	61	8.93
	Don’t Know/Not Sure	52	7.61
Who do you think should decide about the size of the family (how many children to have)?	It is only in the hands of God	255	37.34
	common decision between husband & wife	244	35.72
	It is the woman who should decide	107	15.67
	It is the man who should decide	76	11.13
Who do you think should decide about the interruption of a pregnancy (voluntary abortion)?	Common decision between husband and wife	277	40.56
	It is only in the hands of God	208	30.45
	It is the woman who should decide	125	18.3
	It is the man who should decide	72	10.54
Do you think that married women in your community usually stop using family planning due to fear of conflict with their husbands?	Completely Agree	246	36.02
	Slightly Agree	221	32.36
	Slightly Disagree	87	12.74
	Not sure	70	10.25
	Strongly Disagree	37	8.35

**Table 4:** Multiple regression analysis to identify the factors associated with not using FPs among pastoralist communities.

Variables	Model 1: Demographic Factors	Model 2: Model 1 + Fertility	Model 3: Model 2 + Access to Health Services	Model 4: Model 3 + Attitude of Women and RH Rights	Model 5: Model 1 to Model 4	Final Model: Model 1 but Only Significant and Those with Better Fit
Women’s demographic factors						
Region (ref: Afar)						
SNNP	1.58 [044, 5.69]					
Oromia	1.47 [0.31, 6.91]					
Districts (ref: Afambo)						
Assayita	0.47 [0.22, 0.98] *	10.9 [0.55, 222]				0.66 [0.24, 1.7]
Dasenech	0.17 [0.04, 0.68] *	0.33 [0.01, 5.97]				0.96 [0.11, 8.55]
Hammer	-	0.63 [0.12, 3.22]				14 [1.6, 123]
Arero	-	110 [4.4, 2733]				4.28 [0.77, 23]
Women age (ref: 15–24)						
25–34	1.2 [0.64, 2.24]					
35–49	1.2 [0.5, 2.5]					
Maternal education (ref: less than primary)						
Secondary and above	0.14 [0.0, 0.3] ***	0.05 [0.005, 0.53] *	0.48 [0.09, 2.4]			
Religion (ref: Christian)						
Muslim	1.08 [0.28, 4.22]					0.89 [0.14, 5.5]
Nonbeliever	1.36 [0.48, 3.7]					0.94 [0.3, 2.8]
Traditional	0.44 [0.16, 1.17]					0.16 [0.04, 0.57] **
Polygamy (ref: no)						
Yes	0.36 [0.2, 0.64] ***	2.2 [0.63, 7.7]				
Occupation (ref: student)						
Employed	1.68 [0.95, 2.98]	1.02 [0.26, 3.9]				
Farmer	0.28 [0.09, 0.85]	2.5 [0.09, 67.4]				
Source of income (ref: animal husbandry)						
Land cultivation	0.88 [0.51, 1.51]	1.27 [0.36, 4.4]				
Others (including daily labour, own business, employed)	0.28 [0.09, 0.855] *	3.88 [0.43, 34.57]				
Current working status (ref: no)						
Yes						
Expenditure	0.83 [0.51, 1.36]					0.90 [0.9, 0.9] **
Income	0.99 [0.99, 1.00]					0.11 [0.013, 0.93] *
Fertility-related factors						
Birth spacing (≥)						
Less 2		3.34 [1.15, 9.6] **	4.66 [2.2, 9.7] ***	3.08 [1.24, 7.66] *	3.1 [1.4, 6.5] **	2.9 [1.48, 5.7] **
Wanted pregnancy for index child (ref: yes)						
No		6.8 [1.73, 26.9] ***	4.39 [1.73, 11.2] **	16.7 [4.6, 60] ***	9.3 [3.2, 26] ***	7.7 [3.44, 17] ***
Access to and use of FP services						
Source of information (ref: health facility)						
Health extension workers (HEWs)		0.3 [0.03, 2.4]				
Community Volunteer		0.78 [0.09, 6.2]				
Others—Radio/TV/peers		0.29 [0.02, 3.01]				
Reason for not using (ref: distance to health facility)						
Fear of infertility		2.89 [0.44, 18.8]				
Not allowed in my religion		17.2 [1.22, 244]				
Want to get pregnant		1.22 [0.25, 5.8]				
Others		1.28 [0.27, 6.04]				
Level of Satisfaction (ref: satisfied)						
Not satisfied			4.17 [1.9, 9.16] ***	10.09 [3.1, 32] ***	6.23 [2.4, 15] ***	
Information method (ref: health provider)						
HEW			0.12 [0.02, 0.6]			
Community meeting			0.33 [0.06, 1.7]			
Health development agent			0.34 [0.06, 1.8]			
Attitudes towards FP and reproductive rights of women						
Open discussion (ref: yes)						
No			2.2 [1.01, 4.8] *	1.4 [0.4, 5]		
Perception of prevention pregnancy (ref: prevention of pregnancy good for family)						
No, preventing a pregnancy is not good for the family				0.02 [0.0, 0.15] **	0.10 [0.0, 0.5] ***	
Yes, it is good because of other reasons				0.07 [0.01, 0.54] **	0.15 [0.03, 0.7] **	
Attitude of wife using FP on her own (ref: agrees wife should suggest FP to her husband)						
Disagree				1.45 [0.28, 7.3]		
I don’t know/not sure				10.5 [0.34, 320]		
Husband supports using modern contraceptive (ref: yes)						
No				1.08 [0.27, 4.2]		15.8 [7.7, 32] ***
It is normal for a husband to suggest to his wife/girlfriend to use (ref: agree)						
I don’t know/not sure				0.73 [0.013, 39.6]		
Disagree				0.9 [0.16, 5.3]		
If unmarried couples want to have sexual intercourse before marriage, it is normal (ref: Agree)						
I don’t know/not sure				0.29 [0.02, 3.5]		
Disagree				0.72 [0.2, 2.3]		
Perception of whether a women should suggest FP choice to her husband (ref: she doesn’t trust her husband)						
Not good/decent wife				0.46 [0.09, 2.39]		
He is not committed/unfaithful				0.48 [0.08, 2.74]		
He is concerned for his wife’s health/feeding his child				0.37 [0.04, 2.83]		
Man suggests FP choice to his wife (ref: he doesn’t trust his wife)						
Not good/decent husband				0.28 [0.05, 1.49]		
He is not committed/unfaithful				3.09 [0.59, 16.2]		
He is concerned for his wife’s health/feeding his children				3.64 [0.46, 28.6]		
Decision on number of children to have (ref: someone else)						
It is the man who should decide				0.46 [0.05, 3.77]		
It is the woman who should decide				2.25 [0.27, 18.4]		
It should be a common decision between husband and wife				1.47 [0.32, 6.63]		
Who should decide on termination of pregnancy (ref: Someone Else)						
It is the man who should decide				18.32 [1.97, 169.8]	2.7 [0.8, 8.38]	
It is the woman who should decide				1.44 [0.17, 12.08]	1.7 [0.58, 5]	
It should be a common decision between husband and wife				0.45 [0.08, 2.5]	1.1 [0.3, 3]	
Community members expect unmarried women not to use FP methods (ref: agree)						
Not sure/don’t know				1.64 [0.18, 14.29]		
Disagree				1.02 [0.22, 4.6]		
Unmarried women cease using FP method because of fearing disrespect from the community (ref: agree)						
I don’t know/not sure				0.045 [0, 2.19]		
Disagree				0.6 [0.12, 2.8]		
Unmarried women can use FP method if the community receives adequate health education (Comm Health education) (ref: disagree)						
Agree				3.39 [0.79, 14.4]		
Married women are not allowed to use FP method unless she gets approval from her husband (ref: agree)						
I don’t know/not sure				4.30 [0.08, 221]	1.3 [0.28, 6]	
Disagree				5.17 [1.15, 23.2]	3.0 [1.16, 8] **	
Most husbands highly expect their wives not to use FP service so as to have large family size (ref: agree)						
I don’t know/not sure				0.65 [0.013, 30.65]		
Disagree				0.65 [0.14, 2.9]		
If married women use FP method without the consent of their husband, conflict will arise, even ending in divorce (ref: agree)						
I don’t know/not sure				1.61 [0.05, 50]		
Disagree				0.65 [0.15, 2.71]		
Married women aged less than 18 years can use FP services (ref: agree)						
Disagree				3.5 [1.17, 10.5]	2.4 [0.99, 5.8] *	
It is mainly the woman’s responsibility to ensure contraception is used regularly (ref: agree)						
Disagree					1.28 [0.45, 3.6]	2 [1.01, 3.9] **
A man will decide if he uses condoms when having sex with his partner (ref: agree)						
Disagree					0.61 [0.16, 2.2]	
A man should never allow his wife to decide on the size of the family (how many children to have) (ref: agree)						
Disagree					1.06 [0.41, 2.69]	
A couple should decide together if they want to have children (ref: agree)						
Disagree					0.68 [0.23, 2]	
Decision on purchase of large household items (ref: both husband and wife)						
Husband or someone else					0.51 [0.22, 1.15]	0.17 [0.07, 0.4] **
Decision on Healthcare for women (ref: both husband and wife)						
Husband or someone else					2.8 [1.03, 7.6] **	3.54 [1.4, 8.5] **

* *p* < 0.001; **p < 0.01, *** *p* < 0.05.

## Data Availability

The data presented in this study are available upon request to the corresponding author. The data are not publicly available due to privacy reasons.
